# Better Virological Outcomes Among People Living With Human Immunodeficiency Virus (HIV) Initiating Early Antiretroviral Treatment (CD4 Counts ≥500 Cells/µL) in the HIV Prevention Trials Network 071 (PopART) Trial in South Africa

**DOI:** 10.1093/cid/ciz214

**Published:** 2019-03-16

**Authors:** Geoffrey Fatti, Ashraf Grimwood, Jean B Nachega, Jenna A Nelson, Kelsea LaSorda, Gert van Zyl, Nelis Grobbelaar, Helen Ayles, Richard Hayes, Nulda Beyers, Sarah Fidler, Peter Bock

**Affiliations:** 1 Kheth’Impilo AIDS Free Living, Cape Town, South Africa; 2 Division of Epidemiology and Biostatistics, Department of Global Health, Faculty of Medicine and Health Sciences, Stellenbosch University, Cape Town, South Africa; 3 Department of Epidemiology, University of Pittsburgh Graduate School of Public Health, Pennsylvania; 4 Department of Infectious Diseases and Microbiology, University of Pittsburgh Graduate School of Public Health, Pennsylvania; 5 Department of Epidemiology and International Health, Johns Hopkins University Bloomberg School of Public Health, Baltimore, Maryland; 6 Department of Medicine and Centre for Infectious Diseases, Faculty of Medicine and Health Sciences, Stellenbosch University, Cape Town; 7 Division of Medical Virology, Faculty of Medicine and Health Sciences, Stellenbosch University and National Health Laboratory Service, Tygerberg, South Africa; 8 Anova Health Institute, Paarl, South Africa; 9 Department of Clinical Research, London School of Hygiene and Tropical Medicine, United Kingdom; 10 Zambart, Ridgeway Campus University of Zambia, Lusaka; 11 Department of Infectious Disease Epidemiology, London School of Hygiene and Tropical Medicine, United Kingdom; 12 Desmond Tutu Tuberculosis Centre, Department of Paediatrics and Child Health, Faculty of Medicine and Health Sciences, Stellenbosch University, Cape Town, South Africa; 13 Department of Medicine, Imperial College London and Imperial College National Institute for Health Research Biomedical Research Centre, United Kingdom

**Keywords:** HIV/AIDS, early antiretroviral treatment, virological outcomes, baseline CD4 cell count, HPTN 071 (PopART) Trial

## Abstract

**Background:**

There have been concerns about reduced adherence and human immunodeficiency virus (HIV) virological suppression (VS) among clinically well people initiating antiretroviral therapy (ART) with high pre-ART CD4 cell counts. We compared virological outcomes by pre-ART CD4 count, where universal ART initiation was provided in the HIV Prevention Trials Network 071 (PopART) trial in South Africa prior to routine national and international implementation.

**Methods:**

This prospective cohort study included adults initiating ART at facilities providing universal ART since January 2014. VS (<400 copies/mL), confirmed virological failure (VF) (2 consecutive viral loads >1000 copies/mL), and viral rebound were compared between participants in strata of baseline CD4 cell count.

**Results:**

The sample included 1901 participants. VS was ≥94% among participants with baseline CD4 count ≥500 cells/µL at all 6-month intervals to 30 months. The risk of an elevated viral load (≥400 copies/mL) was independently lower among participants with baseline CD4 count ≥500 cells/µL (3.3%) compared to those with CD4 count 200–499 cells/µL (9.2%) between months 18 and 30 (adjusted relative risk, 0.30 [95% confidence interval, .12–.74]; *P* = .010). The incidence rate of VF was 7.0, 2.0, and 0.5 per 100 person-years among participants with baseline CD4 count <200, 200–499, and ≥500 cells/µL, respectively (*P* < .0001). VF was independently lower among participants with baseline CD4 count ≥500 cells/µL (adjusted hazard ratio [aHR], 0.23; *P* = .045) and 3-fold higher among those with baseline CD4 count <200 cells/µL (aHR, 3.49; *P* < .0001).

**Conclusions:**

Despite previous concerns, participants initiating ART with CD4 counts ≥500 cells/µL had very good virological outcomes, being better than those with CD4 counts 200–499 cells/µL.

**Clinical Trials Registration:**

NCT01900977.

## Teaser

There are concerns that people initiating antiretroviral therapy (ART) with high CD4 counts may exhibit reduced HIV virological suppression. In this study, however, participants with CD4 count ≥500 cells/µL had excellent virological outcomes, better than those with lower CD4 counts.

Effective virological suppression (VS) among people living with human immunodeficiency virus (PLHIV) receiving antiretroviral therapy (ART) is essential for both individual benefit and to reduce human immunodeficiency virus (HIV) transmission [1, 2]. Although there has been progress toward the Joint United Nations Programme on HIV/AIDS (UNAIDS) target of 90% of people receiving ART achieving VS by 2020 [3], a number of challenges remain [4]. Concerns have been expressed that ART adherence and VS may be suboptimal among those starting ART with higher CD4 cell counts as they may be asymptomatic [5, 6]. Indeed, higher CD4 count at ART initiation has previously been associated with increased viremia and virological failure (VF) [7–9].

In 2015, the World Health Organization (WHO) recommended ART initiation for all PLHIV [10]. This recommendation was adopted by South Africa in September 2016, with prior CD4 count initiation thresholds being 350 cells/µL and 500 cells/µL, consistent with international recommendations. As part of the HIV Prevention Trials Network (HPTN) 071 (PopART) cluster-randomized trial, all adults living with HIV have been able to access ART at 3 facilities in South Africa since January 2014 [11], almost 3 years prior to the country’s clinical guidelines revision.

There are very few published data of virological outcomes of PLHIV initiating ART with baseline CD4 count >500 cells/µL in sub-Saharan Africa [12, 13]. Implementation research is needed to assess the effectiveness of the expanded HIV treatment strategy and the provision of ART for HIV prevention [5, 6]. The HPTN 071 (PopART) trial provided a unique opportunity to examine outcomes of people initiating ART irrespective of CD4 count in sub-Saharan Africa prior to routine implementation of the revised WHO clinical guidelines. In view of previous concerns regarding virological response during universal test and treatment for HIV, this study aimed to quantify virological outcomes of people initiating ART early (baseline CD4 count ≥500 cells/µL) and to compare these virological outcomes to people initiating ART with lower CD4 counts in the Western Cape Province, South Africa.

## METHODS

### Setting

A prospective cohort study was conducted among adults initiating ART at 3 public primary healthcare clinics randomly allocated to the full intervention arm of the HPTN 071 (PopART) cluster-randomized trial in South Africa. A full description of the trial design is published elsewhere [11, 14]. Communities surrounding the clinics received household delivery of a combination HIV prevention package, including HIV education and HIV rapid testing in the home by community HIV care providers, referral to the clinics, and active linkage to ART. Study clinics offered ART regardless of CD4 count for all adults living with HIV. Provincial guidelines’ CD4 count ART initiation thresholds during the study period were ≤350 cells/µL until January 2015 and ≤500 cells/µL thereafter [15].

Adults aged **≥**18 years initiating ART between January 2014 and November 2015 with available baseline CD4 count measurements and at least 1 on-treatment viral load (VL) were included in analyses. Clients already receiving ART who transferred into the clinics were excluded. Database closure was 31 January 2017.

### Outcomes

The outcomes were:

1. Proportions of participants with measured VL being elevated (≥400 copies/mL) at 6-month intervals after starting ART to a maximum of 30 months.
2. Cumulative probability of confirmed VF (2 consecutive VLs of >1000 copies/mL) after starting ART.
3. Cumulative probability of viral rebound (VL ≥400 copies/mL) after initial VS, among participants achieving initial VS who had at least 1 repeat VL >30 days after initial VS.

VS was defined as VL <400 copies/mL. The principal exposure was baseline CD4 count, defined as the most recent CD4 count routinely performed within 6 months before starting ART. CD4 counts were categorized as <200, 200–499, and ≥500 cells/μL. Early ART was defined as a baseline CD4 count of ≥500 cells/μL. The category 200–499 cells/μL was the reference category for all regression analyses.

All participants received standard care and routine follow-up as per Department of Health (DOH) ART guidelines excepting that individuals starting ART outside of DOH guidelines signed informed consent. All participants received standard of care adherence support provided by clinic-based counselors complemented by support from community HIV care providers who were scheduled to see clients a minimum of annually. Guidelines recommended that VL testing be performed 4–6 months after starting ART and at month 12, then annually; when elevated (≥400 copies/mL), the VL was to be repeated 2–6 months later.

### Data Collection and Analysis

Clinical data were extracted from sites’ electronic ART patient databases, electronic tuberculosis (TB) registers, and National Health Laboratory Services databases. Baseline characteristics of adults included and excluded from analyses due to absent VL data within the primary exposure categories of interest (baseline CD4 count ≥500 cells/μL and 200–499 cells/μL) were compared using Wilcoxon rank-sum or Pearson χ^2^ tests as appropriate. Baseline characteristics of the included study sample were compared across strata of baseline CD4 cell count. To estimate relative risks of an elevated VL, crude and adjusted modified Poisson regression with generalized estimating equations to account for clustering by individual was used, with an exchangeable correlation structure [16]. Separate models were constructed for the first year of ART and for the period after the first year to 30 months. All models were controlled for time on ART and healthcare facility. The Wald test was used for global hypothesis tests of the overall effect of multilevel categorical predictor variables.

Kaplan-Meier estimates were used to analyze time to confirmed VF after starting ART, time to viral rebound after initial VS, and time from starting ART to initial VS. Time was measured from the date of starting ART (or date of first VS for viral rebound analyses) to either the event of interest otherwise censored at the most recent available VL result or 24 months following entry, whichever occurred last. The log-rank test was used to compare exposure categories. Cox proportional hazards regression was used for crude and adjusted analyses of predictors of confirmed VF and viral rebound. To account for clustering of observations within facilities, stratified Cox regression was conducted allowing the baseline hazard for each facility to vary. Proportional hazards assumptions were confirmed to be not violated using scaled Schoenfeld residuals.

For the main regression analyses, multiple imputation of missing covariate values using chained equations was conducted assuming missing covariate values were missing at random [17, 18], using 10 imputed datasets. The proportions of missing covariate values that were imputed were low: baseline WHO clinical stage, 1.0%; choice of first nucleoside reverse transcriptase inhibitor drug, 1.1%; choice of nonnucleoside reverse transcriptase inhibitor (NNRTI) drug, 1.1%. Baseline covariates eligible for inclusion in all multivariable models were age, sex, WHO stage, pregnancy, concurrent TB, initial ART regimen, prior ART exposure, and year of starting ART.

As a sensitivity analysis to assess any effect due to missing outcome data, missing VL results were also imputed with fully conditional specification for both included individuals and those excluded from the main analyses due to absent VL data [19]. VL data were imputed as binary values (suppressed/elevated) according to the guidelines’ recommended testing intervals (6, 12, and 24 months after starting ART) for the duration that individuals remained in care at the site during follow-up. Multivariable regression of factors associated with an elevated VL was then repeated on the expanded dataset using the same set of predictor variables included in the main analyses. Statistical analyses were performed with Stata version 15.1. Ethical approval was provided by the Stellenbosch University Health Research Ethics Committee.

## RESULTS

During the enrollment period, 2864 individuals started receiving ART, of whom 1901 met inclusion criteria and were included in analyses (Supplementary Figure 1). The proportion of individuals excluded due to absent VL data did not differ by baseline CD4 count strata (Supplementary Table 1). No statistically significant differences in baseline characteristics were found between excluded and included individuals within baseline CD4 count strata, except that excluded individuals had a higher proportion who initiated ART in 2015 (Supplementary Table 2).

Among included participants, the median baseline CD4 count was 331 cells/µL (Table 1). The distribution of participants by baseline CD4 count strata was 477 (25.1%), 1024 (53.9%), and 400 (21.0%) with CD4 count <200, 200–499, and ≥500 cells/µL, respectively. Participants who initiated early ART (baseline CD4 count ≥500 cells/µL) had less advanced baseline WHO clinical stage disease, were less likely to be receiving concomitant TB treatment, and had an appreciably lower proportion of males than participants in lower CD4 count strata. Those with baseline CD4 count <200 cells/µL had a higher proportion with prior ART exposure.

**Table 1.** Baseline Characteristics of Study Participants According to Strata of Baseline CD4 Cell Count (N = 1901)

Characteristic	Baseline CD4 Count Category, Cells/μL			All Participants
	<200	200–499	≥500	
No. included (row %)	477 (25.1)	1024 (53.9)	400 (21.0)	1901 (100)
Baseline CD4 count, cells/μL, median (IQR)	113 (61–158)	341 (268–407)	623 (552.9–766)	331 (199–471)
Age, y, median (IQR)	33.7 (29.3–39.7)	31.7 (26.4–37.8)	31.0 (26.2–37.9)	32.1 (27.1–38.7)
Age category, y				
18–24	43 (9.0)	187 (19.3)	80 (20.0)	310 (16.3)
25–34	222 (46.5)	488 (47.7)	189 (47.3)	899 (47.3)
35–49	187 (39.2)	268 (26.2)	99 (24.8)	554 (29.1)
≥50	25 (5.2)	81 (7.9)	32 (8.0)	138 (7.3)
Male sex	201 (42.1)	313 (30.6)	82 (20.5)	596 (31.4)
Baseline WHO stage (n = 1883)				
I/II	310 (65.8)	887 (87.4)	364 (91.7)	1561 (82.9)
III	133 (28.2)	115 (11.3)	30 (7.6)	278 (14.8)
IV	28 (5.9)	13 (1.3)	3 (0.8)	44 (2.3)
Pregnant	12 (2.5)	57 (5.6)	33 (8.3)	102 (5.4)
Baseline TB treatment	112 (23.5)	75 (7.3)	20 (5.0)	207 (10.9)
First NRTI in ART regimen (n = 1880)				
Tenofovir	452 (96.6)	1002 (98.8)	396 (99.5)	1850 (98.4)
Zidovudine	7 (1.5)	7 (0.7)	2 (0.5)	16 (0.9)
Stavudine	9 (1.9)	5 (0.5)	0 (0)	14 (0.7)
NNRTI in ART regimen (n = 1881)				
Efavirenz	457 (98.1)	1005 (98.7)	395 (99.5)	1857 (98.7)
Nevirapine	9 (1.9)	13 (1.3)	2 (0.5)	24 (1.3)
Previous ART exposure for >3 mo	25 (5.2)	15 (1.5)	4 (1.0)	44 (2.3)
Year of starting ART				
2014	125 (26.2)	312 (30.5)	141 (35.3)	578 (30.4)
2015	352 (73.8)	712 (69.5)	259 (64.8)	1323 (69.6)

Data are presented as no. (%) unless otherwise indicated.

Abbreviations: ART, antiretroviral therapy; IQR, interquartile range; NNRTI, nonnucleoside reverse transcriptase inhibitor; NRTI, nucleoside reverse transcriptase inhibitor; TB, tuberculosis; WHO, World Health Organization.

The proportions having available VL results among those remaining in care were high (80%–100%) at all 6-monthly time-points after initiating ART; these did not differ by baseline CD4 count category excepting at 18 months (which was not a recommended time-point for VL testing according to the clinical guidelines) (Supplementary Table 3).

Time to initial VS was shorter with increasing baseline CD4 count: After 12 months, the cumulative probability of VS was 79.7%, 82.9%, and 85.8% among participants with baseline CD4 counts <200, 200–499, and ≥500 cells/µL, respectively (log-rank *P* for trend = .005).

**Figure 1.**
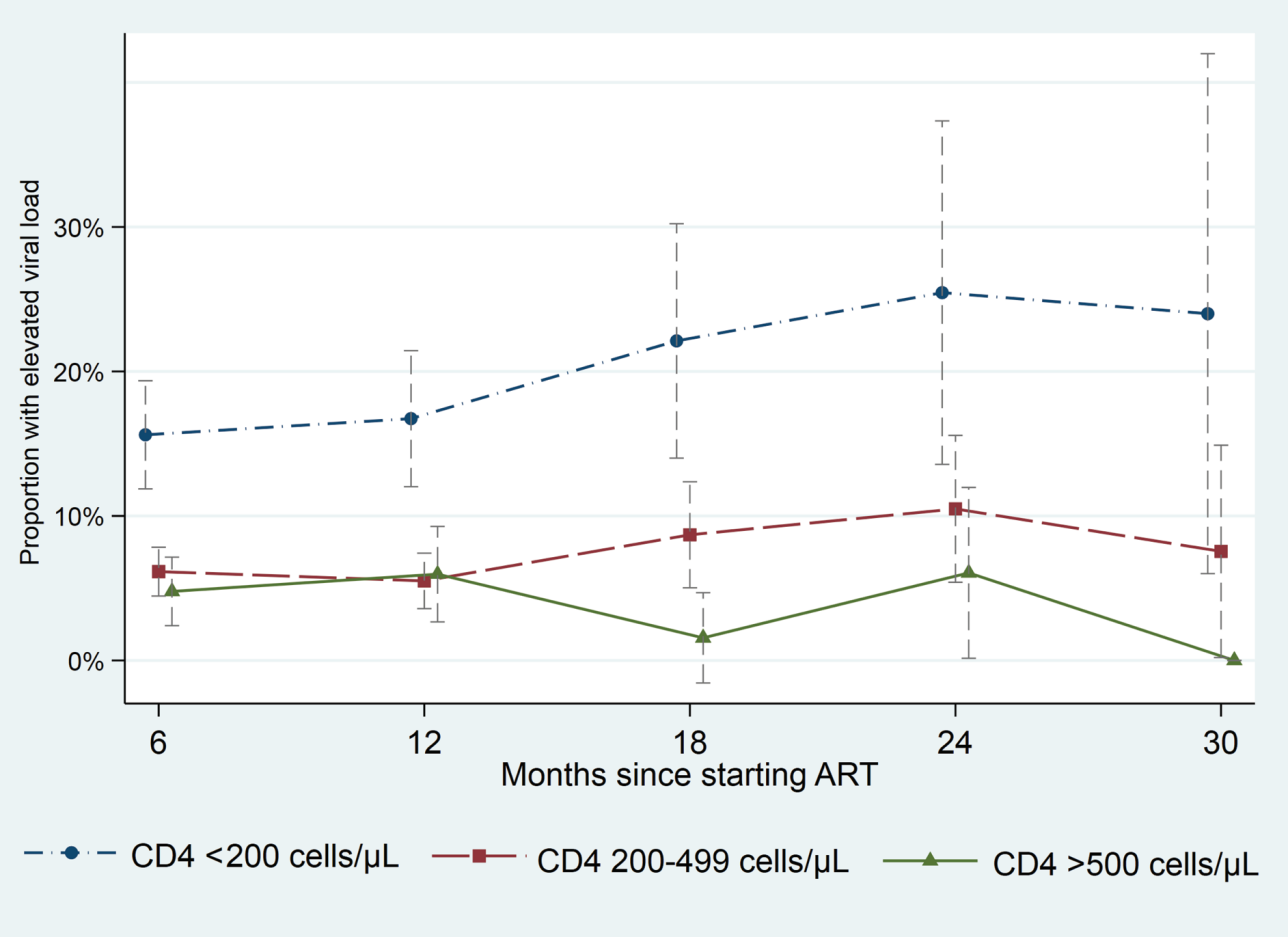
Proportion of participants with elevated viral load (>400 copies/mL) according to baseline CD4 cell count strata and time on antiretroviral therapy (ART). Vertical bars are 95% confidence intervals.

Between 6 and 30 months of ART, VS was higher according to baseline CD4 count strata: 82.2% (653/794), 93.3% (1635/1752), and 95.2% (634/666) among participants with baseline CD4 counts <200, 200–499, and ≥500 cells/µL, respectively (*P* for trend < .0001). VS was ≥94% at all 6-monthly time-points among participants with baseline CD4 count ≥500 cells/µL up to 30 months. Illustrated in Figure 1, participants with baseline CD4 count <200 cells/µL had substantially increased proportions with elevated VLs, whereas participants with baseline CD4 count ≥500 cells/µL had lower proportions with elevated VLs between months 18 and 30, compared to participants with baseline CD4 count 200–499 cells/µL.

Table 2 shows predictors of an elevated VL. During the first year of ART, there was no difference in the risk of an elevated VL between participants with baseline CD4 count ≥500 cells/µL vs 200–499 cells/µL. However, between months 18 and 30, participants with baseline CD4 count ≥500 cells/µL had a substantially reduced risk of an elevated VL compared to participants with baseline CD4 count 200–499 cells/µL (3.3% vs 9.2%, respectively; *P* = .020), with a 70% reduction in multivariable analyses (adjusted relative risk [aRR], 0.30 [95% confidence interval {CI}, .12–.74]; *P* = .010). Participants with baseline CD4 count <200 cells/µL had a considerably increased risk of an elevated VL during both the first year on ART (16.1%) and during months 18–30 (23.4%).

**Table 2.** Proportions With an Elevated Viral Load (>400 Copies/mL) and Baseline Predictors of an Elevated Viral Load After Starting Antiretroviral Therapy

	Elevated VL	Months 3–12 of ART						Elevated VL	Months 18–30 of ART					
		Crude Analysis			Multivariable Analysis^a^				Crude Analysis			Multivariable Analysis^a^		
Characteristic	No. (%)	RR	(95% CI)	*P* Value	Adjusted RR	(95% CI)	*P* Value	No. (%)	RR	(95% CI)	*P* Value	Adjusted RR	(95% CI)	*P* Value
CD4 category, cells/μL				<.0001			<.0001				<.0001			<.0001
<200	98 (16.1)	2.73	(2.03–3.70)	<.0001	2.21	(1.60–3.07)	<.0001	43 (23.4)	2.56	(1.66–3.93)	<.0001	2.40	(1.52–3.79)	<.0001
200–499	78 (5.9)	Ref	…		Ref	…		39 (9.2)	Ref	…		Ref	…	
≥500	27 (5.2)	0.89	(.56–1.39)	.61	0.96	(.62–1.48)	.84	5 (3.3)	0.27	(.10–.70)	.007	0.30	(.12–0.74)	.010
Age, y				.090			.077				.97			.66
18–24	29 (8.3)	0.82	(.54–1.24)		0.99	(.66–1.48)		13 (10.7)	0.92	(.49–1.71)		1.13	(.61–2.08)	
25–34	103 (8.9)	Ref	…		Ref	…		40 (11.2)	Ref	…		Ref	…	
35–49	65 (9.1)	1.02	(.73–1.41)		0.82	(.58–1.13)		29 (12.2)	1.04	(.62–1.72)		0.78	(.47–1.31)	
≥50	6 (3.3)	0.37	(.16–.86)		0.36	(.16–.82)		5 (10.9)	0.87	(.31–2.37)		1.14	(.42–3.08)	
Sex														
Female	123 (7.3)	Ref	…		Ref	…		58 (10.8)	Ref	…		Ref	…	
Male	80 (10.6)	1.46	(1.40–1.52)	<.0001	1.13	(.84–1.53)	.41	29 (13.0)	1.22	(.77–1.91)	.40	0.30	(.55–1.46)	.67
WHO stage				<.0001			.0001				.020			.80
I/II	132 (6.5)	Ref	…		Ref	…		58 (9.4)	Ref	…		Ref	…	
III	58 (17.5)	2.71	(2.48–2.97)		2.39	(1.60–3.56)		23 (19.7)	2.15	(1.49–3.11)		1.18	(.61–2.30)	
IV	11 (20.4)	3.14	(1.44–6.84)		2.01	(1.06–4.10)		6 (27.3)	2.60	(1.13–5.97)		1.20	(.48–3.02)	
Pregnancy in women														
No	119 (7.6)	Ref	…		…	Ref		57 (11.4)	Ref	…		Ref	…	
Yes	4 (3.2)	0.42	(.13–1.37)	.15	0.48	(.17–1.32)	.156	1 (2.7)	0.18	(.01–1.34)	.095	0.31	(.03–2.35)	.26
Concurrent TB														
No	172 (7.8)	Ref	…		…	Ref		69 (10.1)	Ref	…		Ref	…	
Yes	31 (13.3)	1.72	(1.17–2.51)	.005	0.68	(.42–1.12)	.13	18 (22.0)	2.29	(1.30–3.81)	.001	1.18	(.61–2.26)	.61
First NRTI				.013			.52				<.0001			.0037
Tenofovir	194 (8.1)	Ref	…		Ref	…		78 (10.6)	Ref	…		Ref	…	
Zidovudine	3 (18.8)	2.18	(.55–8.70)		1.51	(.66–3.41)		6 (54.6)	4.90	(2.45–9.79)		3.60	(1.43–9.09)	
Stavudine	5 (25.0)	3.12	(2.30–4.23)		1.31	(.57–3.05)		3 (42.9)	4.54	(1.21–16.92)		2.49	(.84–7.38)	
NNRTI														
Efavirenz	199 (8.3)	Ref	…		Ref	…		84 (11.2)	Ref	…		Ref	…	
Nevirapine	2 (6.5)	0.76	(.41–1.37)	.36	0.64	(.20–2.11)	.47	3 (60.0)	6.40	(3.41– 12.01)	<.0001	1.88	(.64–5.48)	.25
Prior ART exposure														
No	194 (8.1)		…		…	…		82 (11.0)		…		…	…	
Yes	9 (19.2)	2.36	(.98–5.72)	.056	1.43	(.67–3.05)	.34	5 (27.8)	4.00	(1.92–8.40)	< .0001	2.85	(1.41–5.73)	.003
Year starting ART														
2014	70 (8.8)	Ref	…		…	…		64 (13.5)	Ref	…		…	…	
2015	133 (8.0)	0.86	(.60–1.24)	.42	…	…		23 (8.0)	0.79	(.39–1.52)	.49	…	…	

Modified Poisson regression with generalized estimating equations to account for clustering was used, including 10 multiple imputed datasets for missing covariate values. Models were controlled for time on ART and healthcare facility.

Abbreviations: ART, antiretroviral therapy; CI, confidence interval; NNRTI, nonnucleoside reverse transcriptase inhibitor; NRTI, nucleoside reverse transcriptase inhibitor; RR, risk ratio; TB, tuberculosis; VL, viral load; WHO, World Health Organization.

^a^Multivariable models included baseline CD4 cell count category, age, sex, baseline WHO stage, pregnancy, concurrent TB, first NRTI in ART regimen, NNRTI in ART regimen, and prior ART exposure.

**Figure 2.**
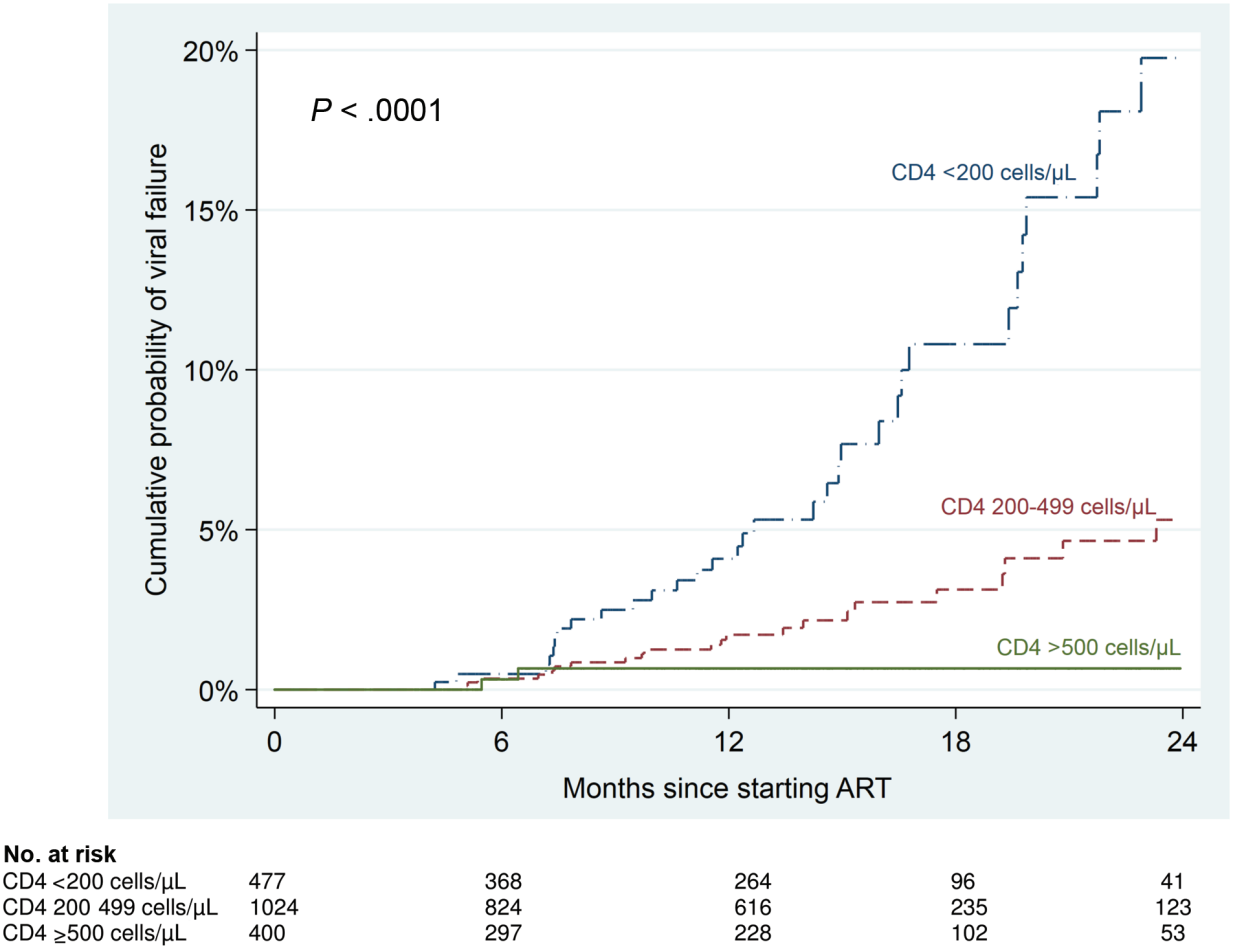
Kaplan-Meier failure estimates of confirmed virological failure (2 consecutive viral loads >1000 copies/mL) according to baseline CD4 count strata after starting antiretroviral therapy (ART).

Sixty (3.2%) participants developed confirmed VF during follow-up, with an incidence rate of 2.9 cases per 100 person-years (PY) (95% CI, 2.2–3.7). VF was inversely related to baseline CD4 count. The incidence rate of VF was 7.0, 2.0, and 0.5 cases per 100 PY among participants with baseline CD4 counts of <200, 200–499, and ≥500 cells/µL, respectively (*P* < .0001). After 24 months, the cumulative probability of VF was 19.8%, 5.3%, and 0.7% among participants with baseline CD4 counts of <200, 200–499, and ≥500 cells/µL, respectively (Figure 2). The cumulative probability of VF among participants with baseline CD4 count >500 cells/µL remained unchanged between months 7 and 24. In multivariable analyses, compared to participants with baseline CD4 count 200–499 cells/µL, those with baseline CD4 count ≥500 cells/µL had a 77% reduced risk of VF (adjusted hazard ratio [aHR], 0.23 [95% CI, .05–.97]; *P* = .045), while those with baseline CD4 <200 cells/µL had a 3-fold increased risk of VF (aHR, 3.49; *P* < .0001) (Table 3).

**Table 3.** Predictors of Confirmed Virological Failure Among Participants Initiating Antiretroviral Therapy

				Crude Analysis			Multivariable Analysis^a^		
Characteristic	Virologic Failure, no./No. (%)	PY	Rate per 100 PY (95% CI)	HR	(95% CI)	*P* Value	Adjusted HR	(95% CI)	*P* Value
Total sample	60/1901 (3.2)	2103.6	2.9 (2.2–3.7)	…	…		…	…	
CD4 count, cells/μL						< .0001			< .0001
<200	35/477 (7.3)	499.6	7.0 (5.0–9.8)	4.10	(2.41–7.00)	< .0001	3.49	(2.00–6.14)	< .0001
200–499	23/1024 (2.2)	1163.2	2.0 (1.3–3.0)	Ref	…		Ref	…	
≥500	2/400 (0.5)	440.8	0.5 (.1–1.8)	0.20	(.05–.84)	.028	0.23	(.05–.97)	.045
Age, y						.10			.29
18–24	6/310 (1.9)	345.4	1.7 (.8–3.8)	0.54	(.23–1.26)		0.75	(.32–1.78)	
25–49	53/1453 (3.6)	1613.8	3.3 (2.5–4.3)	Ref	…		Ref	…	
≥50	1/138 (0.7)	144.4	0.69 (.1–4.9)	0.22	(.03–1.60)		0.22	(.30–1.65)	
Sex									
Female	37/1305 (2.8)	1456.7	2.5 (1.8–3.5)	Ref	…		Ref	…	
Male	23/596 (3.9)	651.9	3.5 (2.3–5.3)	1.40	(.84–2.37)	.196	0.85	(.49–1.48)	.569
Baseline WHO stage						.0001	…		.085
I/II	36/1561 (2.3)	1736.1	2.1 (1.5–2.9)	Ref	…		Ref	…	
III	19/278 (6.8)	298.4	6.4 (4.1–10.0)	3.07	(1.76–5.35)		2.10	(1.07–4.10)	
IV	5/44 (11.4)	52.9	9.5 (3.9–22.7)	3.48	(1.35–8.98)		2.00	(.70–5.71)	
Pregnancy									
Nonpregnant women	36/1203 (3.0)	1341.1	2.7 (1.9–3.7)	Ref	…		…	…	
Pregnant woman	1/102 (1.0)	110.6	0.9 (.1–6.4)	0.33	(.05–2.41)	.275	…	…	
Baseline TB treatment									
No	47/1694 (2.8)	1886.0	2.5 (1.9–3.3)	Ref	…		Ref	…	
Yes	13/207 (6.3)	217.7	6.0 (3.5–10.3)	2.46	(1.33–4.54)	.004	0.97	(.45–2.05)	.927
Regimen first NRTI						.71			
Tenofovir	58/1850 (3.1)	2043.7	2.8 (2.2–3.7)	Ref	…		…	…	
Zidovudine	1/16 (6.3)	22.9	4.4 (.6–31.0)	1.28	(.17–9.26)		…	…	
Stavudine	1/14 (7.1)	16.7	6.0 (.8–42.6)	2.15	(.30–15.6)		…	…	
Regimen NNRTI									
Efavirenz	59/1857 (3.2)	2062.5	2.9 (2.2–3.7)	Ref	…		…	…	
Nevirapine	1/24 (4.2)	23.2	4.3 (.9–30.6)	1.72	(.24–12.47)	.591	…	…	
Previous ART exposure									
No	58/1857 (3.1)	2058.9	2.8 (2.2–3.6)	Ref	…		…	…	
Yes	2/44 (4.6)	44.7	4.5 (1.1–17.9)	1.64	(.40–6.74)	.491	…	…	
Year of starting ART									
2014	31/578 (5.4)	893.5	3.5 (2.4–4.9)	Ref	…		…	…	
2015	29/1323 (2.2)	1210.1	2.4 (1.7–3.4)	1.20	(.66–2.12)	.540	…	…	

Abbreviations: ART, antiretroviral therapy; CI, confidence interval; HR, hazard ratio; NNRTI, nonnucleoside reverse transcriptase inhibitor; NRTI, nucleoside reverse transcriptase inhibitor; PY, person-years; TB, tuberculosis; WHO, World Health Organization.

^a^The multivariable model included baseline CD4 cell count category, age, sex, baseline WHO stage, and concomitant TB at baseline. Crude and adjusted models were stratified by site.

A total of 1082 participants achieved VS and had a subsequent VL measurement. Amongst these, 81 (7.5%) developed viral rebound at a rate of 7.4 cases per 100 PY. In multivariable analyses, participants with baseline CD4 count <200 cells/µL had a significantly increased risk of viral rebound, but there was no difference between participants initiating early ART compared to those with baseline CD4 count 200–499 cells/µL (Supplementary Table 4; Supplementary Figure 2).

In the sensitivity analysis including imputed VL data for those with missing VLs and including individuals excluded from main analyses due to absent VL data, the association between a reduced risk of an elevated VL among those with baseline CD4 count ≥500 cells/µL compared to 200–499 cells/µL remained apparent during months 18–30 (aRR, 0.35; *P* = .027; Supplementary Table 5).

## DISCUSSION

Individuals with baseline CD4 count ≥500 cells/µL had excellent virological outcomes; VS and VF were better in this group compared to participants with baseline CD4 count 200–499 cells/µL, and viral rebound was equivalent. This suggests that, despite individuals with CD4 count ≥500 cells/µL being less likely to have HIV-related symptoms, expansion of programs to encourage early ART initiation across sub-Saharan Africa will support achieving the UNAIDS third “90-90-90” target of VS by 2020. The results suggest that adherence is good among those initiating ART with CD4 counts ≥500 cells/µL, which aligns with recently reported adherence outcomes from another universal test and treat trial [20], and is encouraging for the scale-up of ART for all PLHIV.

Previous research of virological outcomes has primarily been conducted among people with baseline CD4 count <500 cells/µL, and has shown mixed results. Most studies found that low baseline CD4 count predicted viremia and VF [21–24]; however, a Ugandan study found that baseline CD4 count ≥250 cells/µL independently predicted persistent viremia [7].

Higher baseline CD4 count has also predicted poorer virological outcomes in 2 previous studies that included participants with baseline CD4 counts >500 cells/µL [8, 9]. In contrast, VS was similar up to 6–12 months of ART in the early vs deferred ART arms of the Strategic Timing of Antiretroviral Therapy and TEMPRANO trials [25, 26] and a recent Brazilian observational study [27].

Reasons for increased VF among those with low baseline CD4 count include low CD4 count being a marker of lower HIV-specific host immune responses and higher baseline VL, which are predictors of subsequent viremia and VF [28–31]. HIV drug resistance may also contribute to VF in this group that had a higher prevalence of previous ART exposure. Drug resistance is increasing in sub-Saharan Africa [32, 33]; thus, expanding drug resistance testing (including pre-ART screening) is important considering the historic use of NNRTI single agents for prevention of mother-to-child transmission, the anticipated rollout of tenofovir-based preexposure prophylaxis, and the transition to dolutegravir-based regimens [34].

Potential mechanisms for improved virological outcomes with baseline CD4 count ≥500 cells/µL include greater host immune responses, lower baseline VL, fewer comorbidities, less concomitant medication, and fewer drug–drug interactions. Importantly, the results mitigate concerns that people in southern Africa who initiate ART during earlier stages of HIV infection when not clinically unwell will have reduced adherence [5, 6].

It is possible that previous studies that found less advanced immunosuppression to be associated with poorer virological response had lower proportions of participants having prior ART exposure (thus possible drug resistance) among those with lower baseline CD4 counts compared to our study, with subsequently better virological response in this group compared to our study, explaining differences in results to our study. Our study is among the first to measure longer-term virological outcomes in a prospective cohort study among people initiating ART with baseline CD4 count >500 cells/µL in high-HIV-prevalence settings.

Increased investment is being made in South Africa to initiate 2 million people on ART over 2 years [35]. This study suggests that 20% of these may have baseline CD4 counts ≥500 cells/µL, and that their virological outcomes are potentially favorable. Nevertheless, one-quarter of participants had baseline CD4 counts <200 cells/µL; therefore, efforts to identify and link people to ART early in the disease course remain essential, along with screening and prophylaxis for cryptococcal disease and TB and diagnosis and treatment of severe bacterial infections. The median baseline CD4 count in this cohort (331 cells/μL) was, however, substantially higher than historical levels [36], promisingly suggesting that the rollout of ART regardless of CD4 count will lead to increases in baseline CD4 count and associated improved clinical outcomes.

Study strengths include that data were part of a high-quality routine dataset, strengthened by prospective data quality improvement during the HPTN 071 (PopART) trial. Participant follow-up continued for up to 24–30 months. There were high rates of completeness in key data fields with only a small proportion (6.6%) of eligible clients excluded due to missing baseline CD4 counts. VL test completion of those in care was high (>80%), which is notable as routine VL monitoring in other southern African countries is frequently unavailable [37]. Clinic activities were closely aligned to standard care during the study period, supporting generalizability of findings. Study limitations include that a relatively large number of people were excluded from analyses due to absent VL data. Most of these individuals discontinued follow-up during the initial months of treatment prior to initial VL testing, as commonly occurs in southern Africa [38]. The proportions excluded due to absent VL data did not differ by baseline CD4 count strata, and baseline characteristics of excluded and included individuals did not differ significantly within baseline CD4 count strata, excepting for year of ART initiation, which was not associated with any outcomes. Also, the sensitivity analysis including imputed VL data for those excluded due to absent VLs showed similar results to the main analysis, so it is not likely that sampling bias altered outcome comparisons between baseline CD4 count categories. VL at ART initiation may be associated with on-treatment virological outcomes; however, baseline VL data were not included as baseline VLs were not performed as part of the study and are not performed routinely in South Africa. Although participants with baseline CD4 count ≥500 cells/µL were less likely to be receiving concomitant TB treatment and had a lower proportion of males than participants in lower CD4 count strata, these differences are unlikely to have biased outcome measures as neither factor was associated with any of the outcomes in multivariable analyses.

## CONCLUSIONS

The HPTN 071 (PopART) trial has provided a unique opportunity to examine virological outcomes during universal test and treatment for HIV prior to routine implementation. Participants initiating ART with CD4 count ≥500 cells/µL had better (or at least equivalent) VS and reduced VF compared to participants initiating ART with CD4 counts 200–499 cells/µL. These findings provide support for and are encouraging for universal ART implementation in Africa. Expansion of programs targeting early ART initiation are important to reach the UNAIDS second and third “90-90-90” elements of ART coverage and VS. Further research assessing methods to improve VS among those starting ART with CD4 counts <200 cells/µL is warranted, and increased efforts to reduce early ART discontinuation are needed.

## Supplementary Data

Supplementary materials are available at *Clinical Infectious Diseases* online. Consisting of data provided by the authors to benefit the reader, the posted materials are not copyedited and are the sole responsibility of the authors, so questions or comments should be addressed to the corresponding author.

ciz214_Suppl_Supplementary_Material

## Notes

***Financial support.*** This work was supported by the NIAID (cooperative agreements UM1-AI068619, UM1-AI068617, and UM1-AI068613), with funding from PEPFAR. Additional funding is provided by 3ie with support from the Bill & Melinda Gates Foundation, as well as by NIAID, NIDA, and NIMH, all part of the US National Institutes of Health (NIH). J. B. N. is supported by research grants from the NIAID/NIH; the AIDS Clinical Trial Group as member of the Scientific Agenda Steering Committee and senior HIV investigator within the Stellenbosch University Clinical Trial Unit (grant number 2UM1AI069521-08); and the Pittsburgh-Stellenbosch University AIDS-comorbidities Training Research Program (Pitt-SU AICoTRP; NIH/Fogarty International Center; grant number 1D43TW010340-01).

***Acknowledgments.*** The authors acknowledge the PopART study team in South Africa, including Kheth’Impilo, Desmond Tutu TB Centre, Anova Health Institute, the City of Cape Town, and Western Cape Government Department of Health, as well as the National Health Laboratory Service. The authors also thank HPTN 071 (PopART) partners in the United States and the United Kingdom (HPTN, FHI 360 North Carolina, London School of Hygiene and Tropical Medicine, Imperial College, and Zambart).

***Potential conflicts of interest.*** H. A. has received personal fees from Global Fund. G. Z. has received an honorarium from ViiV Healthcare. All other authors report no potential conflicts of interest. All authors have submitted the ICMJE Form for Disclosure of Potential Conflicts of Interest. Conflicts that the editors consider relevant to the content of the manuscript have been disclosed.
