# Supplementary material for: Better Virological Outcomes Among People Living With Human Immunodeficiency Virus (HIV) Initiating Early Antiretroviral Treatment (CD4 Counts ≥500 Cells/µL) in the HIV Prevention Trials Network 071 (PopART) Trial in South Africa
Source: Clin Infect Dis. 2019 Mar 16;70(3):395–403. doi: 10.1093/cid/ciz214 (PMC7768744; doi:10.1093/cid/ciz214)
Supplement: ciz214_Suppl_Supplementary_Material [file ciz214_suppl_supplementary_material.pdf]

Supplementary figure 1: Flow diagram of individuals included and excluded from analyses

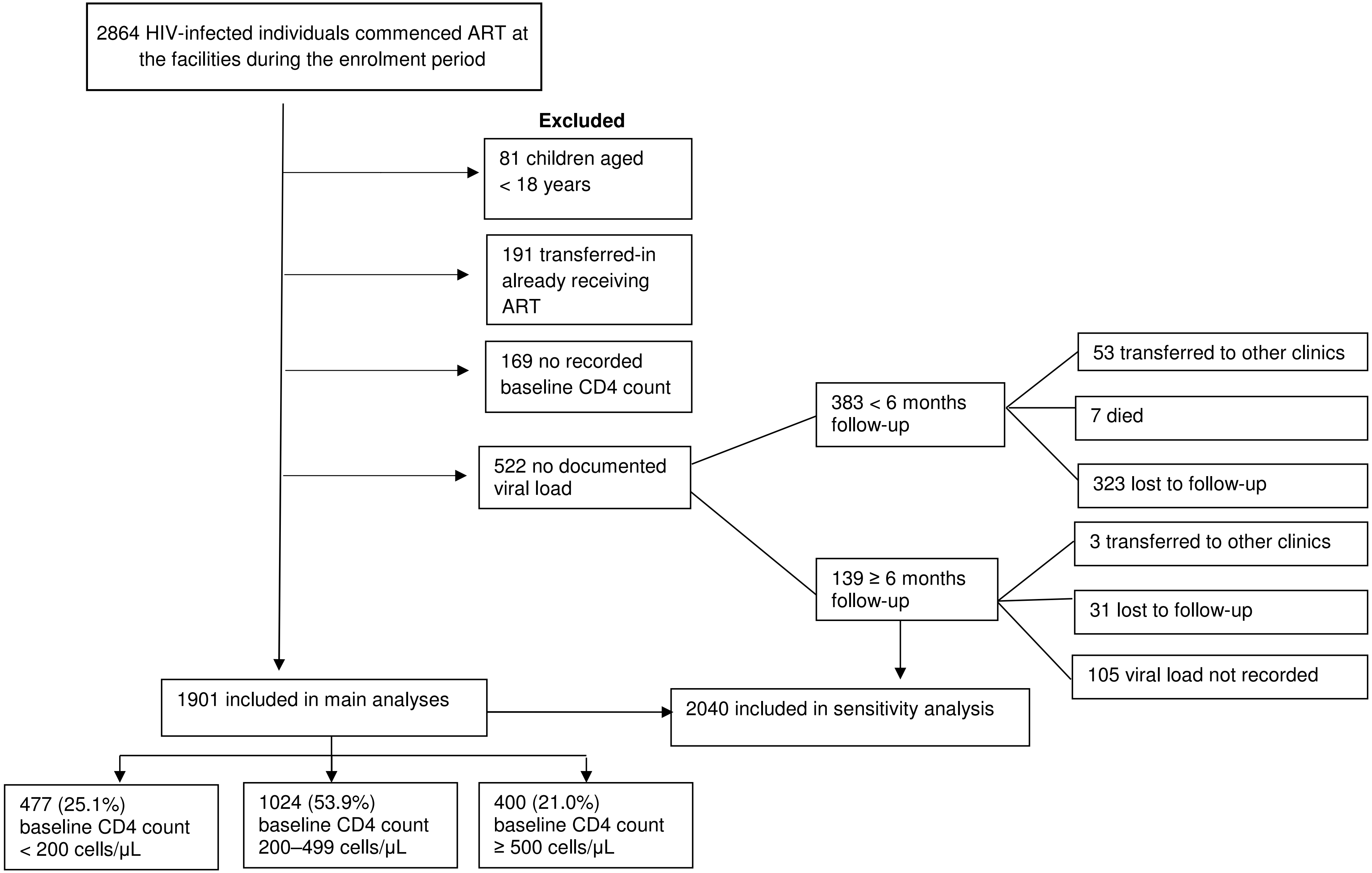

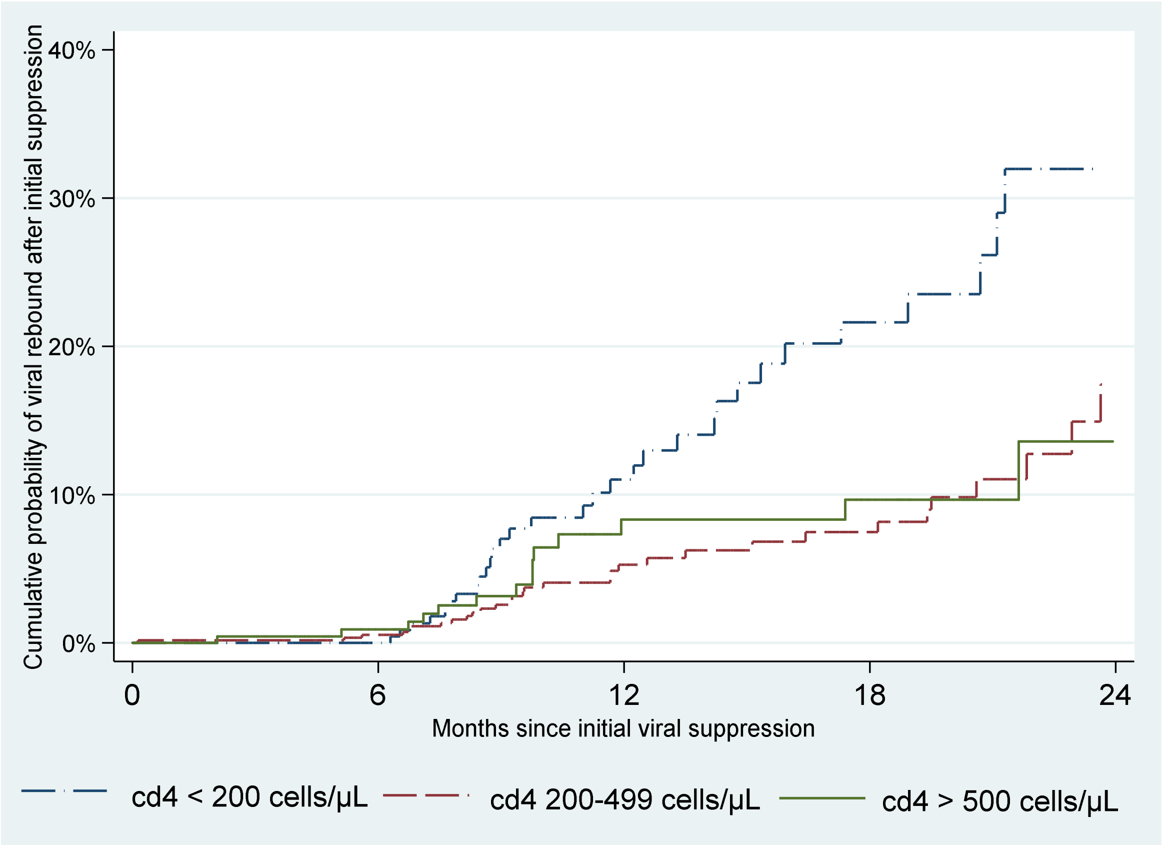

Supplementary figure 2: Kaplan-Meier failure estimates of viral rebound following initial viral suppression by baseline CD4 count strata and time since initial viral suppression.

Supplementary table 1: Comparison of individuals excluded due to absent viral load data by baseline CD4 cell count[1]

	Baseline CD4 cell count			P-value
	**< 200 cells/µL**	**200–499 cells/µL**	**≥ 500 cells/µL**	
**Excluded due to absent VL data, n (%)** (n=2423)	147 (23.5)	269 (20.8)	106 (20.9)	0.36
**If excluded,[2] discontinued care at site during follow-up[3], n (%)** (n=522)	117 (79.6)	214 (79.6)	86 (81.1)	0.94
**If excluded,[2] discontinued care at site during first 6 months of follow-up[3], n (%)** (n=522)	109 (74.2)	193 (71.8)	81 (76.4)	0.63

1. Amongst individuals with recorded pre-ART CD4 cell count.

2. Excluded due to absent viral load data

3. Includes transfer-out to other sites.

VL-viral load

Supplementary Table 2: Comparison of baseline demographic and clinical characteristics according to CD4 count category of adults included and excluded due to absent viral load data[1]

Baseline characteristic	CD4 count 200–499 cells/µL				CD4 count ≥ 500 cells/µL			
	**Excluded** (n=269)	**Included** (n=1024)	**Total** (n=1293)	**P-value**	**Excluded** (n=106)	**Included** (n=400)	**Total** (n=506)	**P-value**
**Male, n (%)**	94 (34.9)	313 (30.6)	407 (31.5)	0.17	17 (16.0)	82 (20.5)	99 (19.6)	0.30
**Age, n (%)**				0.13				0.32
18–34 years	193 (71.8)	675 (65.9)	868 (67.1)		79 (74.5)	269 (67.3)	348 (68.8)	
35–49 years	61 (22.7)	262 (25.6)	323 (25.0)		21 (19.8)	96 (24.0)	117 (23.1)	
≥ 50 years	15 (5.6)	87 (8.5)	102 (7.9)		6 (5.7)	35 (8.8)	41 (8.1)	
**CD4 cell count, cells/µL, median (IQR)**	337 (263–405)	341 (268–407)	339 (268–407)	0.45	616.5 (540–756)	623 (552–766)	622 (547–763)	0.74
**WHO stage, n (%)**				0.20				0.64
I/II	223 (83.5)	887 (87.4)	1110 (86.6)		97 (91.5)	364 (91.7)	461 (91.7)	
III	41 (15.4)	115 (11.3)	156 (12.2)		9 (8.5)	30 (7.6)	39 (7.8)	
IV	3 (1.1)	13 (1.3)	16 (1.3)		0 (0)	3 (0.8)	0 (0.6)	

**Pregnant, n (%)**	22 (8.2)	57 (5.6)	79 (6.1)	0.069	16 (15.1)	33 (8.3)	49 (9.7)	0.083
**TB treatment**	22 (8.2)	75 (7.3)	97 (7.5)	0.63	6 (5.7)	20 (5.0)	26 (5.1)	0.78
**Year of starting ART**				0.003				0.002
2014	57 (21.2)	312 (30.5)	369 (28.5)		21 (19.8)	141 (35.3)	162 (32.0)	
2015	212 (78.8)	712 (69.5)	924 (71.5)		85 (80.2)	259 (64.8)	344 (68.0)	
**Previous ART exposure**	2 (0.7)	15 (1.5)	17 (1.3)	0.36	1 (0.9)	4 (1.0)	5 (1.0)	0.96

1. Amongst those with available baseline CD4 count ≥ 200 cells/μL

IQR-interquartile range; WHO-World Health Organization; TB-tuberculosis; ART-antiretroviral treatment

Supplementary table 3: Participants remaining in care who received viral load testing at six-monthly intervals after initiating antiretroviral treatment by baseline CD4 count category

Months after ART initiation		Baseline CD4 count category (cells/µL)			P-value
		< 200	200–499	≥ 500	
6	Remaining in care, n	451	976	375	
	Viral load done, n (%)	365 (80.9)	781 (80.0)	314 (83.7)	0.3
12	Remaining in care, n	254	553	216	
	Viral load done, n (%)	245 (96.5)	515 (93.1)	201 (93.1)	0.15
18	Remaining in care, n	104	255	102	
	Viral load done, n (%)	104 (100)	210 (82.4)	64 (62.7)	<0.0001
24	Remaining in care, n	55	143	66	
	Viral load done, n (%)	55 (100)	143 (100)	66 (100)	1
30	Remaining in care, n	25	53	21	
	Viral load done, n (%)	25 (100)	53 (100)	21 (100)	1

ART-antiretroviral treatment

Supplementary Table 4: Predictors of viral rebound (>400 copies/ml) following initial viral suppression to < 400 copies/ml

	Viral rebound, n (%)	Person-years	Crude Analysis			Multivariable analysis[a]		
			HR	95% CI	P-Value	Adjusted HR	95% CI	P-Value
**CD4 count category**								
< 200 cells/μL	36 (13.9)	257.7	2.57	1.55–4.25	<0.0001	2.37	1.38–4.09	0.002
200 – 499 cells/μL	31 (5.2)	596.7	Reference			Reference		
≥ 500 cells/μL	14 (6.1)	234.2	1.09	0.58–2.06	0.792	1.19	0.62–2.26	0.59
**Age**								
18-24 years	14 (7.8)	173.1	1.13	0.63–2.04	0.66	1.27	0.69–2.34	0.43
25-49 years	63 (7.7)	844 .9	Reference			Reference		
≥ 50 years	4 (5.1)	70.6	0.85	0.31–2.37	0.76	0.80	0.27–2.26	0.70
**Gender**								
Female	56 (7.4)	760.2	Reference			Reference		
Male	25 (7.7)	328.5	1.06	0.67–1.71	0.81	0.98	0.58–1.66	0.95
**Baseline WHO stage**								
I/II	59 (6.4)	916.7	Reference			Reference		

III	20 (14.8)	141.6	1.90	1.11–3.24		1.26	0.62–2.55	0.51
IV	2 (8.3)	25.0	0.94	0.22–4.01		0.40	0.71–2.27	0.30
**Pregnancy**								
Non-pregnant women	55 (7.9)	702.7	Reference			Reference		
Pregnant women	1 (1.7)	57.4	0.25	0.62–1.62	0.98	0.27	0.04–1.99	0.20
**Baseline TB treatment**								
No	68 (6.9)	990.6	Reference			Reference		
Yes	13 (13.3)	97.8	1.95	1.06–3.58	0.030	1.25	0.57–2.78	0.57
**Regimen first NRTI**								
Tenofovir	74 (7.1)	1058.4	Reference			Reference		
Zidovudine	4 (50.0)	8.9	4.91	1.73–13.8	0.003	2.14	0.48–9.57	0.32
Stavudine	2 (25.0)	8.1	4.17	1.01–17.2	0.049	4.00	0.80–19.8	0.090
**Regimen NNRTI**								
Efavirenz	77 (7.3)	1067.3	Reference			Reference		
Nevirapine	3 (23.1)	9.6	7.93	2.32–27.08	0.001	4.15	0.66–26.0	0.13
**Prior ART exposure**								
No	76 (7.2)	1063.2	Reference			Reference		

Yes	5 (18.5)	25.3	2.75	1.07–7.06	0.035	2.34	0.04–1.98	0.20
**Year of starting ART**								
2014	41 (10.1)	574.1	Reference					
2015	40 (5.9)	514.5	1.44	0.76–2.74	0.26			

[a] The multivariable model included baseline CD4 count category, age, gender, baseline WHO stage, pregnancy, concomitant tuberculosis, regimen first NRTI, regimen NNRTI, and prior ART exposure. Crude and adjusted models were stratified by site.

TB–tuberculosis; HR-hazard ratio; WHO–World Health Organization, NRTI–Nucleoside reverse-transcriptase inhibitor, NNRTI-Non-nucleoside reverse–transcriptase inhibitors; ART-antiretroviral treatment

Supplementary table 5. Sensitivity regression analysis of the association between baseline CD4 count and an elevated viral load on antiretroviral treatment when including imputed outcome (viral load) data.[1]

	Months 3 – 12 of antiretroviral treatment						Months 18 – 30 of antiretroviral treatment					
	Crude analysis			Multivariable analysis[2]			Crude analysis			Multivariable analysis[2]		
	RR	95% CI	P-Value	Adjusted RR	95% CI	P-Value	RR	95% CI	P-Value	Adjusted RR	95% CI	P-Value
**CD4 count category (cells/µL)**			<0.0001			<0.0001			<0.0001			0.0001
< 200	2.30	1.74–3.04	<0.0001	1.88	1.39–2.54	<0.0001	2.42	1.59–3.67	<0.0001	2.07	1.34–3.18	0.001
200 – 499	Ref			Ref			Ref			Ref		
≥ 500	0.70	0.46–1.08	0.109	0.77	0.51–1.17	0.21	0.31	0.12–0.76	0.011	0.35	0.14–0.89	0.027

[1] Missing viral load data were imputed for individuals included in the main analyses and those excluded from main analyses due to absent viral load data. Viral load data were imputed for the duration that individuals remained in care at the site during follow-up. All models were controlled for time on ART and healthcare facility.

[2] Multivariable models included baseline CD4 count category, age, gender, baseline WHO stage, pregnancy, concurrent tuberculosis, first NRTI in ART regimen, NNRTI in ART regimen, and prior ART exposure.

RR-relative risk
